# Feasibility of opportunistic dental diagnostics in routine photon-counting CT examinations of the cervical spine

**DOI:** 10.1186/s12903-025-07585-9

**Published:** 2026-01-10

**Authors:** Stefan Sawall, Joscha Maier, Sinan Sen, Holger Gehrig, Ti-Sun Kim, Christian H. Ziener, Heinz-Peter Schlemmer, Stefan O. Schoenberg, Matthias F. Froelich, Marc Kachelrieß, Maurice Ruetters

**Affiliations:** 1 Division of X-Ray Imaging and CT, German Cancer Research Center (DKFZ), Im Neuenheimer Feld 280, Heidelberg, 69120 Germany; 2 Medical Faculty, Heidelberg University, Im Neuenheimer Feld 672, Heidelberg, 69120 Germany; 3 Department of Orthodontics, University Hospital of Schleswig-Holstein, Arnold-Heller-Straße 3, Kiel, 24105 Germany; 4 Department of Operative Dentistry, University Hospital Heidelberg, Heidelberg University, Im Neuenheimer Feld 400, Heidelberg, 69120 Germany; 5 Department of Radiology, German Cancer Research Center (DKFZ), Im Neuenheimer Feld 280, Heidelberg, 69120 Germany; 6 Department of Radiology and Nuclear Medicine, University Medical Centre Mannheim, Heidelberg University, Theodor-Kutzer-Ufer 1-3, Mannheim, 68167 Germany

**Keywords:** Incidental findings, X-Ray Computed Tomography, Opportunistic Diagnostics, Photon-Counting CT

## Abstract

**Background:**

This study evaluates the potential of routine clinical photon-counting computed tomography (PCCT) of the cervical spine for opportunistic dental diagnostics.

**Methods:**

Thirty-three patients undergoing routine PCCT were included in this study, with imaging performed at an average dose of 13 mGy. Images were reconstructed to a voxel size of 156 μm and a slice thickness of 0.4 mm. Quantitative image quality was assessed using the contrast-to-noise ratio (CNR) between dental structures, while qualitative assessment of structures like enamel, dentine, root canals, and cortical bone was conducted by two experienced readers using a five-point scale.

**Results:**

The inter-reader reproducibility and intra-class correlation coefficient were excellent (all > 0.947). CNRs ranged from 1.6 to 6.1 for all relevant contrasts, and qualitative scores were excellent for all dental structures. Dental pathologies were detected in a significant portion of patients: caries-induced decay in 9 patients (27%) at 26 sites, apical lesions in 12 patients (36%) at 26 sites, and alveolar bone loss in 12 patients (36%) at 12 sites. At least one pathology was present in 23 patients (70%).

**Conclusions:**

PCCT offers potential for opportunistic dental diagnostics, providing earlier detection of dental issues and potentially improving overall dental care outcomes.

## Background

Photon-counting computed tomography (PCCT) is a milestone in computed tomography technology, offering significant improvements in terms of spatial resolution, soft tissue contrast, and radiation dose efficiency compared to conventional energy-integrating (EI) systems [1–3]. In case of such conventional systems, an incoming x-ray photon interacts with a scintillator, typically Gadolinium oxysulfide (Gd_2_O_2_S). This results in the emission of optical photons that are guided towards photo-diodes using reflecting lamellae where the desired signal is eventually formed. In novel photon-counting systems, a semiconductor, typically Cadmium telluride (CdTe), replaces the scintillator. The absorption of an x-ray photon results in the emission of a charge cloud that is transported to electrodes or pixels, respectively, using an applied bias voltage. Consecutive high-speed electronics allow for the counting of single photons and a quantification of their energy [4]. Usually, photons are sorted into two to four distinct energy bins, facilitating dual- or even multi-energy acquisitions. To account for the high x-ray flux rates in clinical CT of up to 10^6^ photons/s/mm^2^, the used electrodes are significantly smaller compared to conventional EI systems allowing for a higher spatial resolution or a dose reduction if such a high spatial resolution is not required [5, 6].

Given the demonstrated, favourable properties of photon-counting CT systems in cardiac, muscolo-skeletal and oncologic imaging [7–14], one of the emerging applications of PCCT is in the field of dental imaging [15–21]. Currently, dental cone beam CT (CBCT), sometimes also referred to as digital volume tomography (DVT), is the method of choice for three-dimensional imaging in dentistry [22]. However, recent studies have already investigated the potential of photon-counting computed tomography (PCCT) for dental imaging. These studies have demonstrated that PCCT provides excellent image quality when visualizing dental structures, pathologies, as well as structures of the periodontium. In particular, an early work using an experimental, whole-body photon-counting CT system illustrated the capabilities of this novel imaging modality for the visualization of relevant dental structures (16). A study using the first clinically approved photon-counting CT system investigated the feasibility of diagnostics in complex endodontic tasks and found that fine endodontic structures can be visualized using clinical PCCT [15]. Using the same system, a significant reduction of administered radiation dose and increase in image quality compared to conventional dental CBCT acquisitions was demonstrated [17].

Currently, only a limited number of installations of the world’s first clinically approved photon-counting CT systems (Naeotom Alpha, Siemens Healthineers, Forchheim, Germany), are available. These systems are usually restricted to university hospitals and research facilities. However, it is expected that the number of installed systems will dramatically increase in the next years given that all vendors conduct research into this novel technology and at least have prototype systems readily available [23–25]. Besides dedicated dental examinations, a potential future application of such systems might be opportunistic dental examinations in clinical routine, i.e. in all acquisitions covering the teeth and jaws. For example, about 11.4% or 1.37 Million, respectively, of the approximately 12 Million annual CT examinations in Germany cover at least parts of the teeth and structures of the periodontium. Examples include but are not limited to computed tomography angiography examinations of the carotid arteries (approx. 1.5%, about 180.000 annual examinations) or examinations of the cervical spine (1.8%, 216.000) [26, 27].

An opportunistic approach might maximize the utilization of these imaging data, enhance patient care by detecting dental pathologies and reduce radiation dose. Therefore, this study evaluates the possibility of opportunistic dental diagnostics in routine photon-counting acquisitions of the cervical spine and aims at answering the question whether this novel imaging modality is suitable to visualize a multitude of clinically relevant dental structures and pathologies.

## Methods

### Patient population

A total of 33 adult patients (mean age: 65 ± 17 years) who underwent routine photon-counting computed tomography of the cervical spine or neck without contrast agent using a Naeotom Alpha scanner (Siemens Healthineers, Forchheim, Germany) between May 2023 and May 2024 were retrospectively considered for this study. All patients receiving a corresponding examination within the specified time frame were eligible for inclusion, regardless of the presence of dental restorations such as fillings or implants. Exclusion criteria comprised severe motion artifacts that rendered diagnostic evaluation of the dentition impossible. Consequently, one patient was excluded from the data pool due to pronounced motion artifacts. No other scans showed image degradation that interfered with diagnostic assessment. Minor artifacts, which were predominantly caused by metallic restorations, did not affect the evaluability of the relevant anatomical structures. No cases of complete edentulism were observed. Across all 32 included patients, a total of 683 teeth were available for diagnostic assessment.

The study was conducted in accordance to the Declaration of Helsinki and approved by the Ethics Committee II of the University of Heidelberg (ID 2021 − 659). Informed written consent was obtained from all subjects involved in this retrospective study.

### Image acquisition and reconstruction

A summary of all scan and reconstruction parameters is provided in Table 1. Initial image acquisitions were performed using the stock protocol for cervical spine imaging available at the scanner. The longitudinal scan range extended from approximately the T₁ vertebral level to just above the foramen magnum, encompassing both the maxillary and mandibular arches and thereby fully covering the dentition without protocol modifications for this retrospective study. In clinical routine, the cranial scan limit is typically positioned about 1 cm above the foramen magnum (including a safety margin to ensure visualization of the atlanto‑occipital junction), and patients are scanned supine with a foam pillow under the occiput inducing a cervical spine extension and commonly shifting the teeth into the axial field of view. All acquisitions were performed using a spiral trajectory and using the standard detector mode providing a detector pixel size in the center of rotation of about 300 μm, using a tube voltage of 120 kV, an image quality reference index of 160 and with CARE Dose4D and CARE keV enabled. This resulted in a dose of 13.2 ± 2.3 mGy (CTDI_32 cm_) on average over all patients. For comparison, the diagnostic reference level for CT examinations of the cervical spine is 15.0 mGy in Germany [28] and a conventional high-resolution CBCT acquisitions is in the range of 3–5 mGy (CTDI_16 cm_) [29].

**Table 1:** Scan and reconstruction parameters used in the study presented herein

Trajectory	Spiral
Pitch	1.0
Collimation	144 × 0.4 mm
CT Dose Index (CTDI_32 cm_)	13.2 mGy
Tube Voltage	120 kV
Image Quality Index	160
Reconstruction	QIR(3)
Axial Voxel Size	156 μm
Slice Thickness	400 μm
Slice Increment	200 μm
Kernel	Qr72

Image reconstruction was performed using Quantum Iterative Reconstruction (QIR), the default iterative reconstruction method of the scanner, at strength 3 and using the Qr72 reconstruction kernel (MTF_10%_=17.3 lp/cm). All data were reconstructed to a matrix of 1024 × 1024 voxels covering a field of view (FOV) of 160 mm, resulting in an axial voxel size of 156 μm and using a slice thickness of 0.4 mm and a slice increment of 0.2 mm. The chosen voxel size ensures sufficient sampling of the system’s spatial resolution, as dictated by the Nyquist criterion. Given the MTF_10%_ of 17.3 lp/cm, the smallest resolvable structure is approximately 0.29 mm. To capture this spatial frequency without aliasing, a voxel size below 0.29 mm is required. I.e., the 156 μm voxel size therefore satisfies this condition and preserves the system’s high spatial resolution. This was experimentally confirmed using a dedicated spatial resolution phantom, from which the edge spread function was derived to calculate the line spread function and corresponding MTF. The FOV was placed to enclose the complete upper and lower jaw in all reconstructions.

### Quantitative image analysis

Quantitative image analysis was performed with respect to image noise and contrast-to-noise ratio (CNR). CNR is computed using

1 $$\:\mathrm{C}\mathrm{N}\mathrm{R}=\frac{{f}_{\mathrm{a}}-{f}_{\mathrm{b}}}{\sqrt{{\sigma\:}_{\mathrm{a}}^{2}+{\sigma\:}_{\mathrm{b}}^{2}}}$$

wherein *f*_a_ and *f*_b_ are mean values measured in desired regions-of-interest (ROIs) and *σ*_a_ and *σ*_b_ are the corresponding standard deviations. All used ROIs covered a size of at least 0.5 cm² and were manually placed in dentine, enamel, the cortical bone of the mandible and the masseter muscle, i.e. soft tissue. ROIs were placed in anatomically homologous locations across all subjects whenever possible. Specifically, cortical bone was consistently measured at the inferior border of the mandible at the level of the first molar. Dentine and enamel were assessed in the same designated tooth, preferably the first mandibular molar or the nearest adjacent tooth if not present. Soft tissue was measured centrally within the masseter muscle, avoiding anatomical edges, artifacts, and metallic restorations. To obtain a measure for dose efficiency, the dose-normalized contrast-to-noise ratio (CNRD) is computed as CNRD = CNR/sqrt(D) with D being dose administered in the scan.

### Qualitative image analysis

Qualitative image quality was independently assessed by two calibrated dentists (H.G., M.R.), each with more than 10 years of experience in reporting CBCT scans and 5 years in reporting PCCT scans. The readers analysed the entire stack of slices for each patient using a conventional DICOM viewer (Radiant DICOM Viewer 2023.1, Medixant, Poland). Windowing and levelling was allowed. Image quality with respect to enamel (E), dentine (D), the cemento-enamel-junction (CEJ), root-canals (RC), periodontal space (PS), spongiosa (S) and cortical bone (CB) was evaluated using a five-point quality scale (1 = excellent, 2 = good, 3 = moderate, 4 = poor, 5 = not visible) similar to previous investigations [17]. For calibration, the readers analyzed and discussed corresponding structures in 20 different PCCT scans until agreement was reached prior to image review.

Additionally, the investigators jointly examined potential pathologies in the following categories that would require further evaluation by a dentist: caries, apical osteolyses, and alveolar bone loss. Alveolar bone loss was defined as vertical bone destruction more than 4 mm from the CEJ or as horizontal bone destruction in furcation areas.

### Statistical evaluation

Statistical analysis was performed using the R software package (version 4.3.1., R Foundation of Statistical Computing, Vienna, Austria). Descriptive statistics were used to summarize quantitative image quality parameters, including mean and standard deviation of contrast-to-noise ratio (CNR) and dose-normalized CNR (CNRD). Inter-reader agreement was assessed using intraclass correlation coefficients (ICC). The ICC is considered poor for ICC < 0.40, moderate to good for 0.40 < ICC < 0.75 and excellent for ICC > 0.75, similar to the literature [30]. No formal hypothesis testing was performed, as the study design did not include group comparisons or inferential objectives.

## Results

### Quantitative image analysis

Given a radiation dose level of 13.2 ± 2.3 mGy (CTDI_32 cm_) on average over all patients resulted in an average image noise of 45 HU in the masseter muscle, i.e. in soft tissue. The observed CNRs on average over all patients were as follows: dentine and enamel 5.31 ± 1.62, dentine and mandible 1.74 ± 0.54, and 6.13 ± 2.41 between enamel and cortical bone. This corresponds to dose-normalized CNRD values of 1.7 ± 0.81 mGy^− 1/2^ for the contrast between dentine and enamel, 0.65 ± 0.32 mGy^− 1/2^ for dentine and the cortical bone of the mandible and 2.17 ± 1.12 mGy^− 1/2^ for enamel and the cortical part of the mandible.

### Qualitative image analysis

**Fig. 1:**
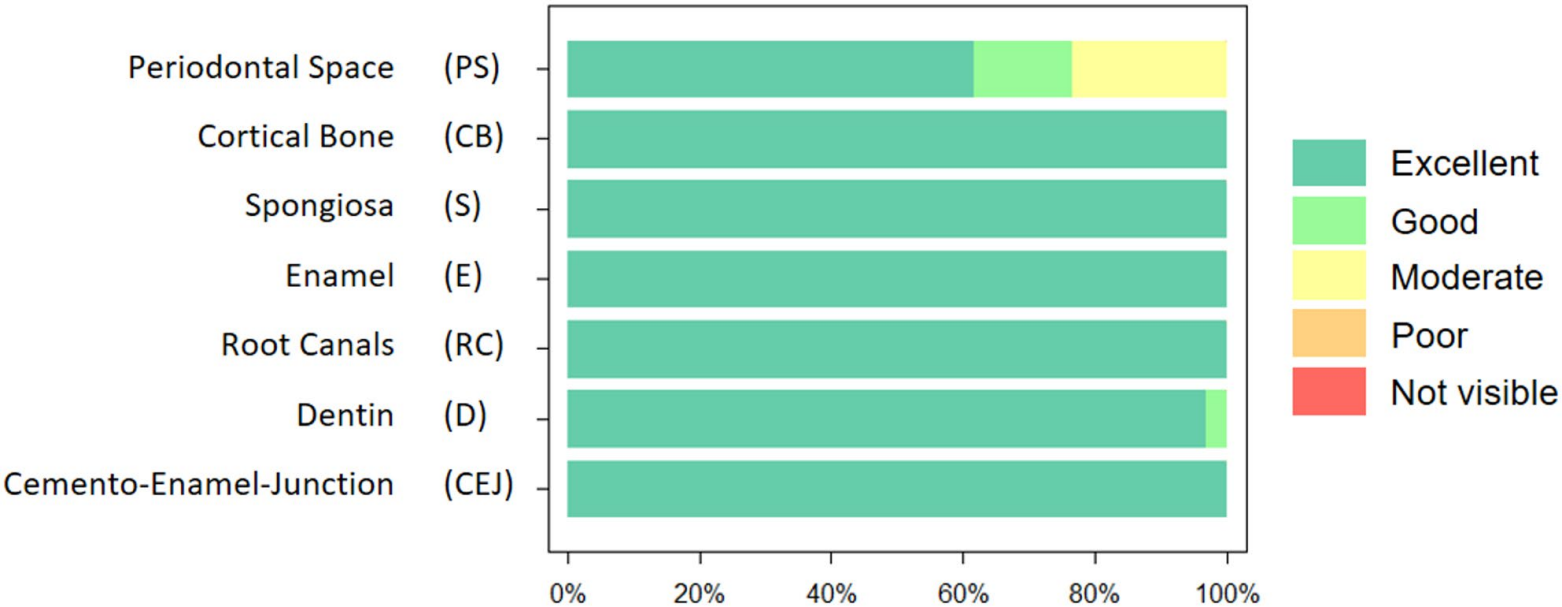
Qualitative imaging scores for the acquisitions assessed by the two readers. In general, most relevant anatomical structures show an excellent rating

In total, the reconstructions contained 683 teeth. Figure 1 illustrates the image quality scores for the structures of interest, as assessed by the two readers. In general, the scores for reconstructions are high across all considered structures, including the periodontal space, cortical bone, spongiosa, enamel, root canals, dentine, and cemento-enamel junction. In particular, both readers rated cortical bone, spongiosa, enamel, root canals and the cemento-enamel-junction excellent over all patients. Only periodontal space and dentine were rated as good or moderate in some patients. No structure was rated as poor in any of the acquisitions. In general, scans showed no image degradation that interfered with diagnostic assessment. Minor artifacts, which were predominantly caused by metallic restorations, did not affect the evaluability of the relevant anatomical structures.

The inter-reader reproducibility was excellent for all structures. In particular, the readers showed an absolute agreement for cortical bone, spongiosa, enamel, root canals and the cemento-enamel-junction. The ratings between both readers only showed slight deviations for periodontal space (ICC = 0.948) and dentin (ICC = 0.976).

Table 2 summarizes the number of potential pathologies identified by the investigators in each category. In total, caries-induced decay was observed in 9 patients (27%) at 26 sites, apical lesions were found in 12 patients (36%) at 26 sites and alveolar bone loss were observed in 12 patients (36%) at 12 sites. In total, at least one of the afore-mentioned pathologies was found in 23 patients (70%).

**Table 2:** Number of dental pathologies detected that require a referral to a dentist

	# patients	# pathologies
Caries decay	9	26
Apical lesions	12	26
Alveolar bone loss	12	12

**Fig. 2:**
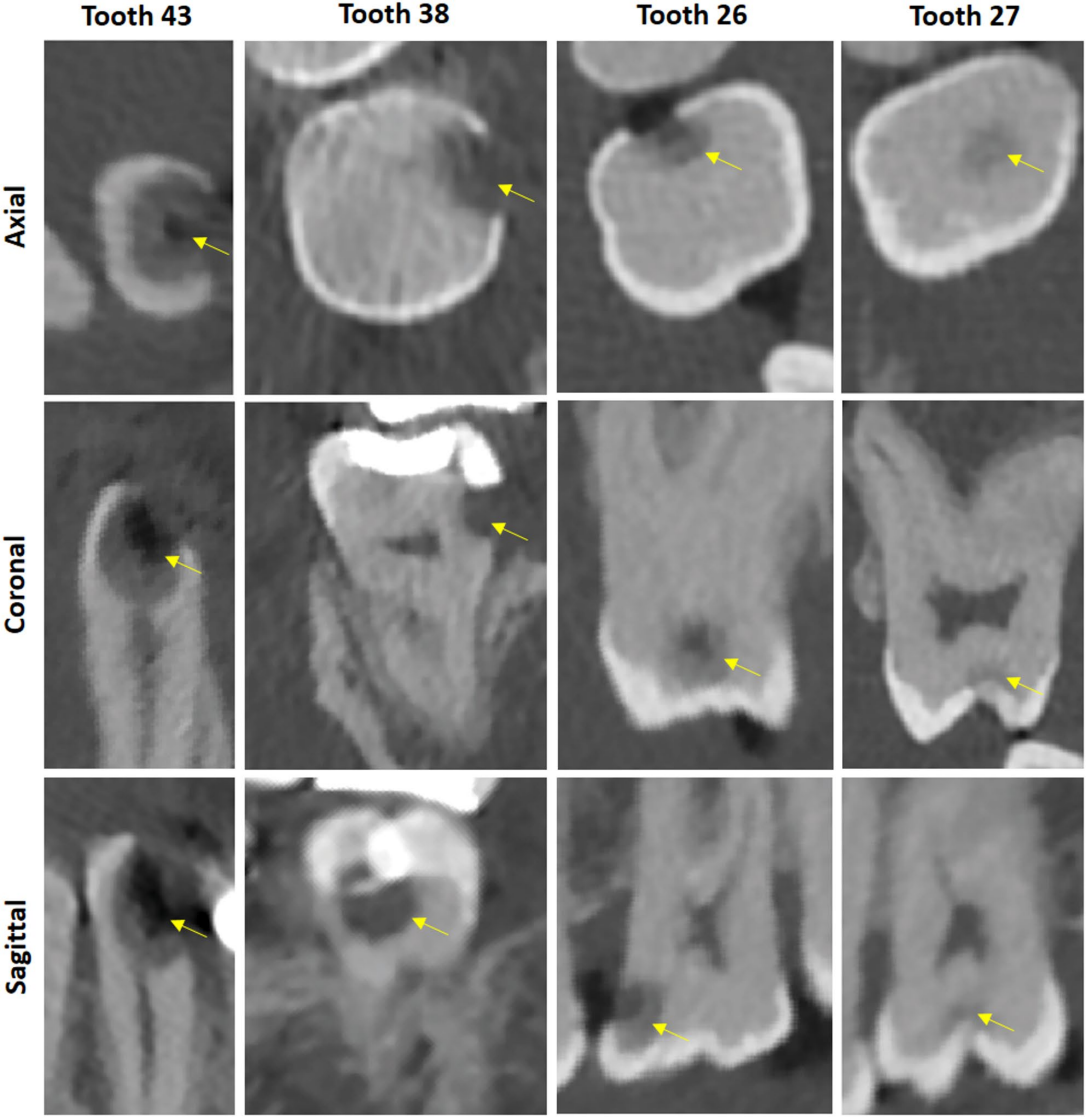
Four exemplary localized carious defects found in four different patients. Yellow arrows indicate the lesions (C = 1300 HU, W = 6000 HU)

**Fig. 3:**
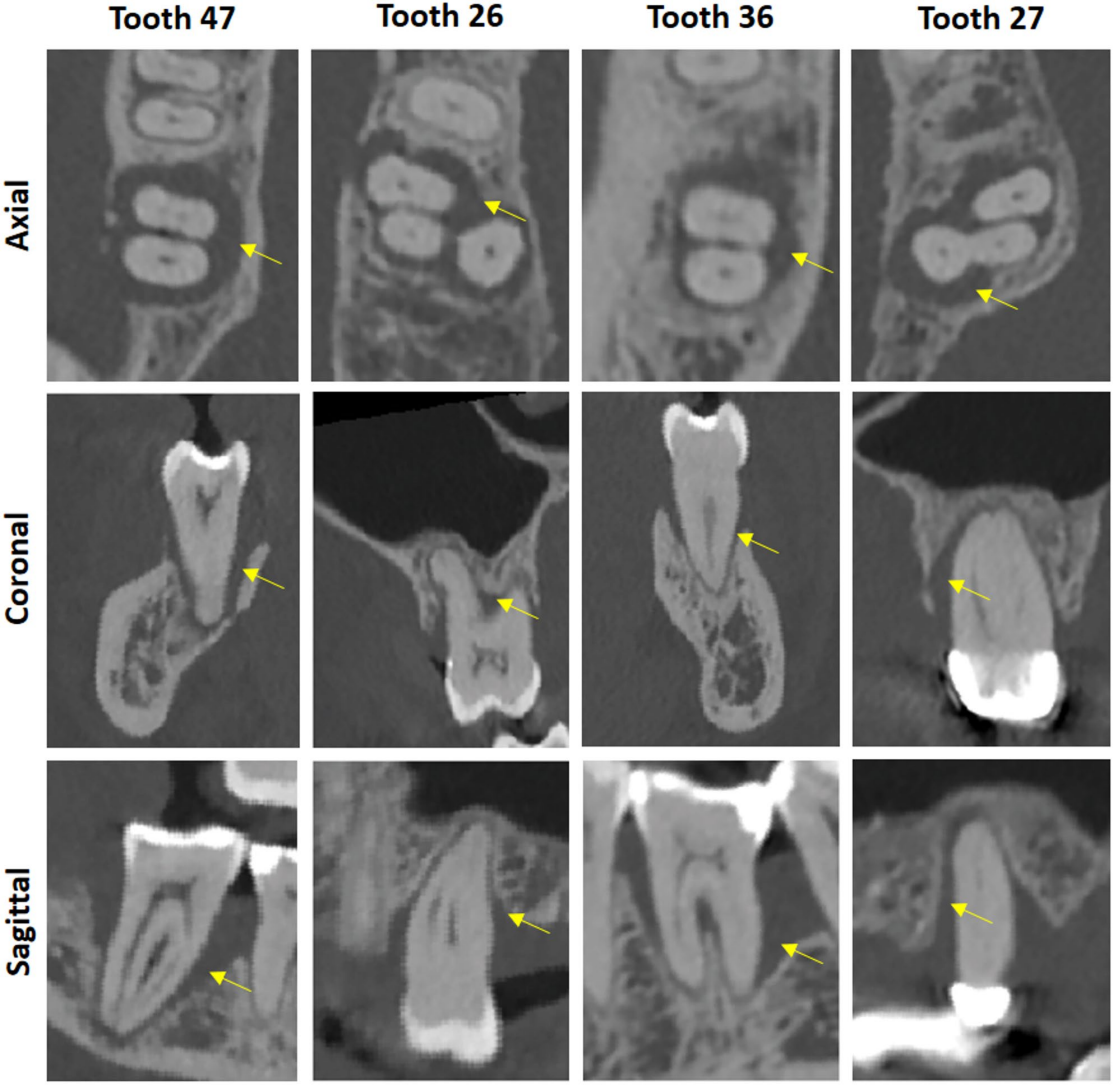
Various periodontal defects found in four different patients. Yellow arrows indicate the periodontal defects. (C = 1300 HU, W = 6000 HU)

**Fig. 4:**
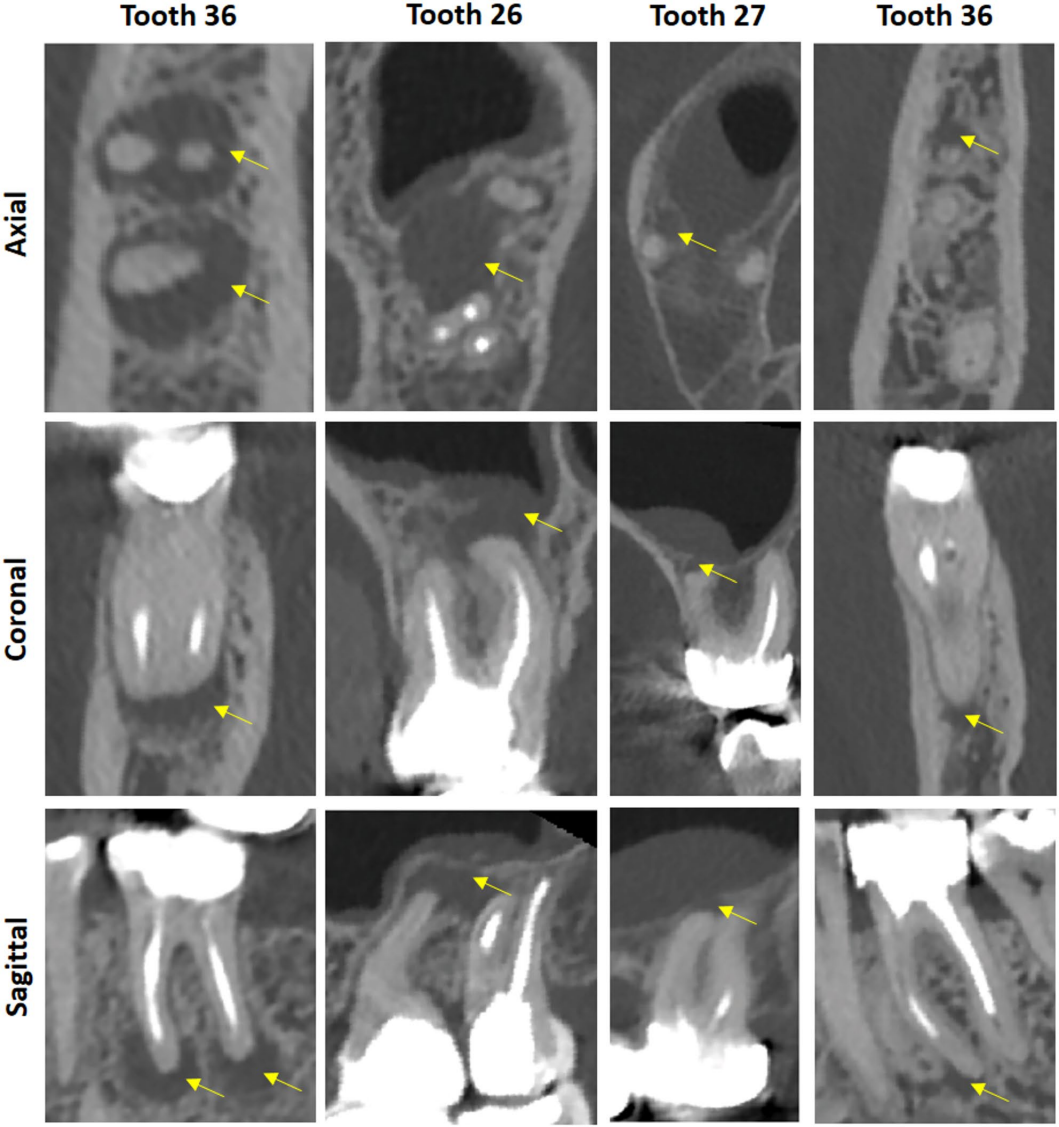
Examples of apical lesions found in the patient data found in four different patients. Yellow arrows indicate the apical lesions (C = 1300 HU, W = 6000 HU)

Figures 2, 3 and 4 show examples for caries-induced decays, apical lesions and alveolar bone loss found among the patients. All the presented pathologies are of dental interest and, from the perspective of opportunistic imaging, require at least a referral for dental care to establish a definitive diagnosis. Note that all tooth numbers in the following correspond to numbers in the FDI notation.

In particular, Fig. 2 depicts several carious defects found in the patients. Tooth 43 in one patient exhibits a widely extended carious defect reaching the root canal, clearly necessitating treatment. Tooth 38 in another patient displays a vestibular carious lesion extending to the middle dentin below a restoration. In all slices, dentin can be well distinguished from enamel. Additionally, the pulp and root canals are also clearly recognizable. Tooth 26 shows a proximal carious lesion preserving the occlusal marginal ridge, extending close to the pulp into the dentin. This also represents a classic dental treatment requirement. Compared to established conventional methods such as periapical and bitewing radiographs, where these lesions are typically detected, this image also allows the practitioner to assess the oro-vestibular extent of the lesion, potentially enabling more precise and potentially minimally invasive therapy. Tooth 27 in another patient demonstrates fissure caries extending from the occlusal surface into the dentin. These are particularly insidious as they are difficult to detect both clinically and in conventional two-dimensional radiographs, especially in the latter case due to the superposition of dentin and enamel in the image.

Figure 3 shows exemplary periodontal defects found in the patients. Tooth 47 of one dataset shows an extensive periradicular defect extending to the root apex of the alveolar bone. This indicates a combined periodontal-endodontic lesion. The mesial and distal extensions are visible, as well as the continuous interradicular bone loss, indicative of a Class III furcation involvement. Additionally, the vestibular bony lamella exhibits a discontinuity. If this were a patient scheduled for, e.g., transplantation, such a radiological finding would likely necessitate extraction. This clear three-dimensional representation of a defect could be crucial for planning necessary periodontal surgical interventions, allowing for more predictable and efficient therapy. Tooth 26 also presents with an extensive periodontal peri- and intraradicular defect of the alveolar bone. This is particularly well observed in the axial view. The vestibular lamella of the alveolar bone also shows a discontinuity. Tooth 36 demonstrates a similar but less extensive defect compared to Tooth 47. Tooth 27 shows an extensive peri- and interradicular bone defect extending to the palatal root apex, clearly visible in the sagittal view. This indicates a combined periodontal-endodontic lesion. Such lesions also require treatment as they can lead to abscess formation and are synonymous with chronic inflammation if left untreated.

Figure 4 shows exemplary apical lesions found in the data. Tooth 36 exhibits an apical lesion on both the mesial and distal roots. This tooth has undergone endodontic treatment. From the perspective of opportunistic imaging, this finding may indicate a dental treatment requirement in the form of either a revision of the endodontic filling or extraction of the tooth, for example, in preparation for a planned jaw irradiation. Teeth 26 and 27 display a communicating apical lesion of the roots, which appears to communicate with the left maxillary sinus. Additionally, the mucosa of the maxillary sinus is swollen. This imaging information indicates a need for dental treatment. Tooth 27 shows an apical lesion on the mesiobuccal root. Furthermore, there is a clearly visible swelling of the mucosa in the vicinity of the maxillary sinus originating from the lesion. Tooth 36 of another patient exhibits an apical lesion on the mesial root. Additionally, in these images, as well as in others, the trabecular bone structures, the cortical bone, and the periodontal ligament space are very clearly visible.

**Fig. 5:**
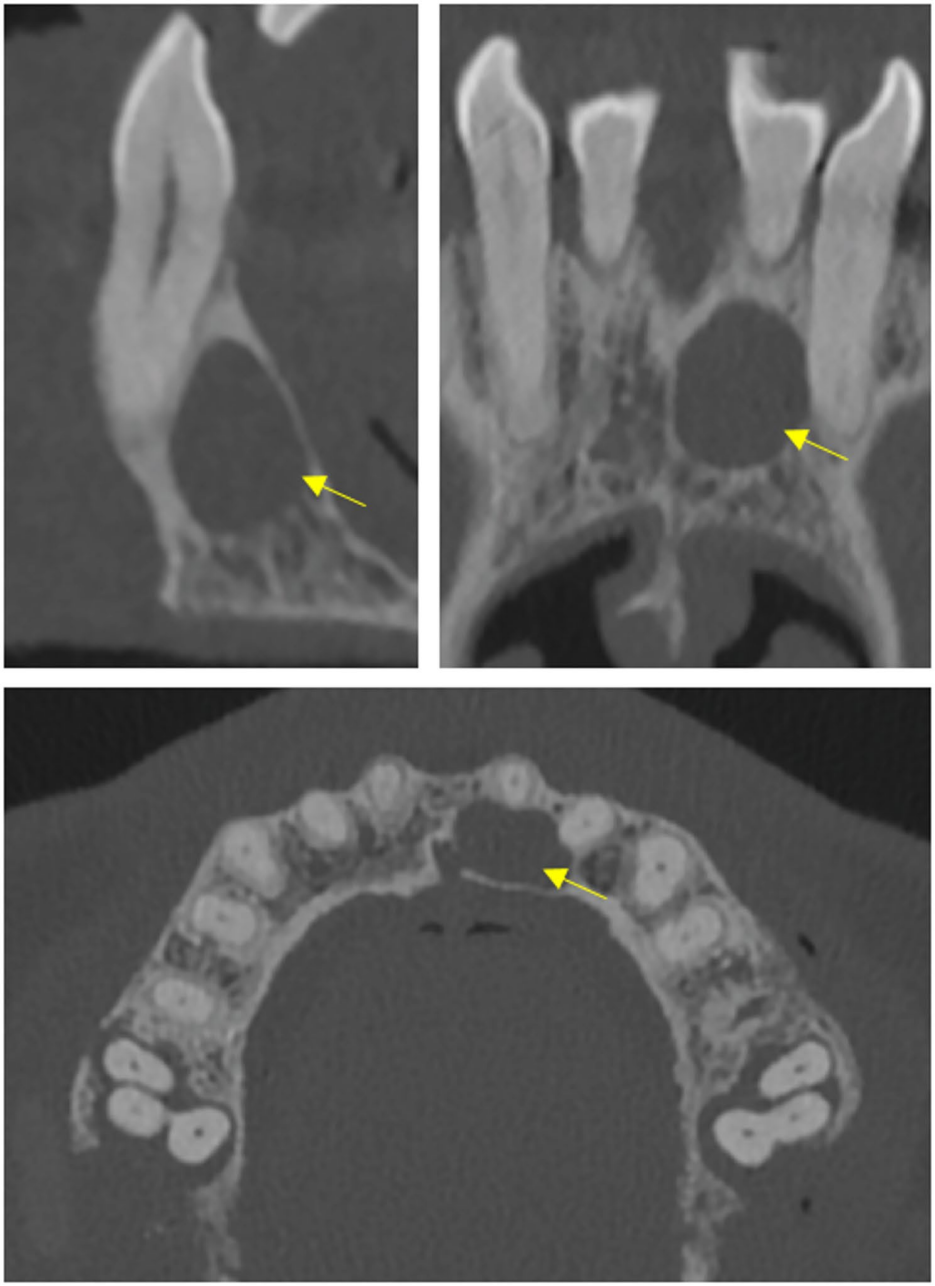
Nasopalatine cyst palatal to the maxillary anterior teeth. Sagittal view (**top left**), frontal view (**top right**) and axial view (bottom). (C = 1300 HU, W = 6000 HU)

Figure 5 shows an incidental finding not covered by the three diagnostic categories discussed previously. Specifically, the image demonstrates a well-circumscribed radiolucent lesion located palatally to the maxillary anterior teeth, with imaging features highly suggestive of a nasopalatine cyst. Such cysts are the most common non-odontogenic cyst in the oral and maxillofacial region. In the coronal reformation, a connection to the incisive foramen is clearly visible, further supporting this radiological impression. However, as no histopathological confirmation was available, this finding should be considered a diagnostic hypothesis. Additionally, periodontal bone defects are visible on teeth 16 and 26.

## Discussion

For the first time, this study investigated whether clinical PCCT could be used to evaluate dental structures and potential pathologies within the context of opportunistic imaging. The hypothesis that this is feasible was confirmed. Both quantitative and qualitative analyses demonstrated excellent results for clinical PCCT.

Quantitatively, the reconstructions show CNR values that are overall high and are comparable to conventional dental CBCT acquisitions reported in previous studies using porcine jaws [17]. However, CNRs are not as high as in the PCCT acquisitions of this previous study. The reason for this is two-fold. On the hand, the previous ex-vivo study only investigated porcine jaws that were devoid of soft tissue. Hence, the intersections lengths were shorter compared to the anatomy of actual patients and hence noise was lower in this study. On the other hand, all reconstructions in the present study have been performed using the standard acquisition mode of the detector. If they were to be acquired using the ultra-high resolution mode, image noise could be further reduced as has been investigated thoroughly before. In particular, prior experiments suggests that the same image quality could be achieved with less than 50% of the dose used herein if the UHR mode were used [5, 6].

Both raters predominantly rated the image quality and delineation of relevant dental structures as excellent. The only major exception was the periodontal space where the quality was slightly lower, though still assessable. These findings are consistent with previously published data from ex vivo studies which also rated the subjective image quality of clinical PCCT as predominantly excellent [16, 17]. Notably, one study, conducted on human specimens used a protocol like in the present study, with a radiation dose level of 38 mGy (CTDI_16 cm_, corresponding to about 18 mGy CTDI_32 cm_) [16]. This protocol, like the present study, showed rating values up to moderate, particularly in the assessment of the periodontal space. This may be attributed to the scanner’s spatial resolution and the fact that the width of the periodontal space is a function of the tooth section, tooth stress and patient age [31, 32]. However, it should be noted that the quality of the depiction was still acceptable from a clinical perspective in both this study and the existing study on human specimens. The existing study also found the quality scores to be on par with high-resolution CBCT [16].

The results regarding the detection of potential dental pathologies that may warrant further clinical evaluation are noteworthy. However, due to the retrospective nature of this study and the absence of dental records or histopathological confirmation, these findings could not be clinically verified thus representing a key limitation of the present work. Accordingly, all diagnoses should be interpreted as radiological hypotheses based on characteristic imaging features. Nevertheless, previous ex vivo research has demonstrated that the detection of apical osteolyses and periodontal bone loss in photon-counting CT is consistent with findings in cone-beam CT [16], supporting the diagnostic potential of this modality for incidental dental assessments. Therefore, it can be assumed that these might indeed represent dental pathologies. Furthermore, the purpose of opportunistic imaging is primarily to alert patients to potential pathologies, with detailed evaluation to follow by a dentist who can correlate these images with clinical findings. Thus, clinical PCCT can potentially reveal previously undetected apical osteolyses. Similarly, asymptomatic carious lesions could be detected, allowing their progression to be halted through appropriate preventive measures. Additionally, periodontitis, often referred to as a “silent disease”, could be detected early. Importantly, this detection is possible in three dimensions, unlike the two-dimensional images commonly used in dental practice. Early detection of dental pathologies can also have significant socioeconomic benefits. For instance, dental emergencies such as dentogenic abscesses could be prevented through earlier opportunistic diagnosis and intervention, reducing potential indirect costs, such as lost workdays, for the general public [33].

To implement opportunistic dental imaging in clinical practice, it is essential to establish appropriate digital infrastructure and interdisciplinary workflows. Although such systems are not yet established in most healthcare environments, including Germany, increasing digitization presents promising opportunities for future integration. A hypothetical, aspirational model for such a workflow could be envisioned as follows: The radiologist performs the PCCT scan, which is stored in a centralized, secure digital environment, such as a cloud-based Picture Archiving and Communication System (PACS), enabling authorized access by other healthcare professionals, including dentists. The radiologist interprets the PCCT data and generates a report that is likewise stored centrally and made available to relevant clinicians. In this process, artificial intelligence (AI) could serve as a supportive tool, for example, by assisting in the detection of dental structures or potential pathologies. However, it is important to emphasize that any AI system used in this context must be specifically trained and validated for dental imaging tasks, and that final interpretation and clinical decision-making must remain the responsibility of qualified radiologists and dentists. Given that such integrated infrastructure is currently unavailable, a more pragmatic interim solution could involve providing patients with their imaging data, e.g., via a digital storage medium, along with a compatible viewer for Digital Imaging and Communications in Medicine (DICOM) images and the radiology report. This enables patients to share the findings with their dentist, thereby bridging the gap between radiology and dental care within the limitations of current healthcare systems.

Still, the potential future integration of artificial intelligence (AI) into PCCT-based dental diagnostics offers promising avenues for future research. Potential applications might include automated detection of caries, periapical lesions, and periodontal bone loss, as well as anatomical segmentation. To ensure reliability, AI tools must be specifically trained and validated on PCCT datasets, given their distinct imaging characteristics compared to CBCT. Future work should focus on dataset development, external validation, and clinical performance assessments to support safe and effective integration into diagnostic workflows.

From a dental perspective, an additional potential benefit of opportunistic PCCT imaging lies in the incidental visualization of root canals. The results of this feasibility study illustrate that detailed in vivo visualization is possible and are consistent with findings from prior ex vivo studies. In clinical practice, root canals may become obliterated due to trauma or progressive dental disease, making them undetectable even in dedicated three-dimensional dental imaging [34]. If historical PCCT scans that include the dentition were available, they could provide valuable anatomical reference points, potentially supporting guided endodontic procedures in complex cases. While promising, these findings require further prospective validation before routine clinical use can be recommended.

## Conclusions

This study represents the first in vivo investigation of opportunistic dental diagnostics using photon-counting CT (PCCT) acquired during routine cervical spine imaging. The results are consistent with previous ex vivo findings and suggest that dental structures, including hard tissues and root canals, can be visualized with high detail. However, these findings are preliminary and based on a small retrospective cohort without clinical or histological confirmation. Therefore, further research is needed to validate diagnostic accuracy, evaluate clinical integration, and assess cost-effectiveness before PCCT can be considered for broader use in dental screening. Nevertheless, this emerging imaging modality shows considerable potential to enhance diagnostic opportunities in incidental dental assessment and to contribute to improved interdisciplinary patient care in the future.

## Data Availability

No datasets were generated or analysed during the current study.

## Abbreviations

AI: Artificial intelligence
CBCT: Cone-Beam CT
CNR: Contrast-to-noise ratio
CNRD: Dose-normalized contrast-to-noise ratio
CT: Computed tomography
CTDI: Computed tomography dose index
DICOM: Digital imaging and communications in medicine
DVT: Digital volume tomography
EICT: Energy-integrating CT
FDI: Fédération Dentaire Internationale
FOV: Field of view
ICC: Intraclass correlation coefficient
MTF: Modulation transfer function
PCCT: Photon-counting computed tomography
ROI: Region of interest
